# The Biological Deserts Fallacy: Cities in Their Landscapes Contribute More than We Think to Regional Biodiversity

**DOI:** 10.1093/biosci/biaa155

**Published:** 2021-01-20

**Authors:** Erica N Spotswood, Erin E Beller, Robin Grossinger, J Letitia Grenier, Nicole E Heller, Myla F J Aronson

**Affiliations:** San Francisco Estuary Institute San Francisco, California in the United States. Erin E. Beller is the Urban Ecology Program manager for the Real Estate and Workplace Services Sustainability Team at Google, Mountain View, California, in the United States; San Francisco Estuary Institute San Francisco, California in the United States. Erin E. Beller is the Urban Ecology Program manager for the Real Estate and Workplace Services Sustainability Team at Google, Mountain View, California, in the United States; San Francisco Estuary Institute San Francisco, California in the United States. Erin E. Beller is the Urban Ecology Program manager for the Real Estate and Workplace Services Sustainability Team at Google, Mountain View, California, in the United States; San Francisco Estuary Institute San Francisco, California in the United States. Erin E. Beller is the Urban Ecology Program manager for the Real Estate and Workplace Services Sustainability Team at Google, Mountain View, California, in the United States; Carnegie Museum of Natural History, Pittsburgh, Pennsylvania, United States; Department of Ecology, Evolution, and Natural Resources, The State University of New Jersey, New Brunswick, New Jersey, United States

**Keywords:** biodiversity conservation, urban biodiversity, cities, regional ecosystems, habitat heterogeneity

## Abstract

Cities are both embedded within and ecologically linked to their surrounding landscapes. Although urbanization poses a substantial threat to biodiversity, cities also support many species, some of which have larger populations, faster growth rates, and higher productivity in cities than outside of them. Despite this fact, surprisingly little attention has been paid to the potentially beneficial links between cities and their surroundings. We identify five pathways by which cities can benefit regional ecosystems by releasing species from threats in the larger landscape, increasing regional habitat heterogeneity and genetic diversity, acting as migratory stopovers, preadapting species to climate change, and enhancing public engagement and environmental stewardship. Increasing recognition of these pathways could help cities identify effective strategies for supporting regional biodiversity conservation and could provide a science-based platform for incorporating biodiversity alongside other urban greening goals.

Cities are embedded in and connected to their surrounding landscapes. Energy, resources, and species all flow across political and geographic boundaries, with impacts on landscape-scale biodiversity. Although urbanization poses a substantial threat to biodiversity (McDonald et al. 2020), cities also support many species, some of which have larger populations, faster growth rates, and higher productivity in cities than elsewhere (Faeth et al. 2011, Bateman and Fleming 2012). Despite this fact, discussion of the implications of ecological links between cities and their surrounding landscapes has focused primarily on the negative impacts, including the export of pollution (Grimm et al. 2008b, Hien et al. 2020) and invasive species (Aronson et al. 2007, Von der Lippe and Kowarik 2008, Bar-Massada et al. 2014, Padayachee et al. 2017), the impacts of domesticated animals on wildlife in adjacent wildland areas (Lepczyk et al. 2004, Metsers et al. 2010, Hanmer et al. 2017), and the potential of cities to create ecological traps (Battin 2004, Sumasgutner et al. 2014, Spear et al. 2018, Tella et al. 2020). However, the successes of some native, nonpest species in cities suggests that we have an incomplete understanding of the full suite of ecological roles cities play within their landscapes and of how positive roles can be bolstered through intentional design. Filling this gap can guide the design and management of urban green spaces to enhance their contributions to regional and global biodiversity conservation. Over the coming decades, as urban footprints grow and the impact of climate change on biodiversity accelerates, we will need cities to contribute to and support global biodiversity conservation.

Cities are unique features, often differing markedly from their surrounding landscapes. They are often located in nonrandom settings with distinctive topographic, edaphic, and hydrologic characteristics, resulting in underlying differences between cities and their surroundings irrespective of urbanization (Luck 2007). Urbanization further modifies the physical landscape and climate, intensifying differences between cities and their surroundings (Grimm et al. 2008b, Pickett et al. 2011, Kaushal et al. 2014). Plant and animal communities are also altered: cities tend to have higher numbers of nonnative species and are often dominated by urbanization-tolerant or synanthropic species (Faeth et al. 2011). Resulting novel species assemblages alter trophic structures and phenologies, which can reduce available resources.

Landscapes surrounding cities also vary widely from relatively intact ecosystems to ecosystems highly modified by intensive or extensive agriculture and plantation forests (Grimm et al. 2008a, Oliveira Hagen et al. 2017). Surrounding landscapes also vary in habitat quality and resource availability depending on the degree of disturbance and habitat homogeneity (Oliveira Hagen et al. 2017, Phillips et al. 2018). Where surrounding landscapes provide high-quality and diverse habitats, there may be little advantage for species to venture into cities. In other cases, extreme disturbance in the surrounding landscape may lead to more resources and opportunities in cities compared with their surroundings. These differences, combined with variation in how species respond to urbanization, lead to large differences in how species use urban landscapes, and what benefits cities may provide.

The unique conditions found in cities have a variety of species-specific impacts that range from negative to neutral to positive depending on each species’ behavioral and life-history characteristics and tolerance to urbanization (Evans et al. 2011, Sol et al. 2014). Although overwhelming evidence suggests that urbanization is a net negative for biodiversity, there are also many informative examples of species that are either neutrally effected or doing well in cities. For example, unique habitat features in cities may support particular species or life history needs, or provide refuge from threats in the surrounding landscape. In addition, there is potential for cities to both increase regional genetic diversity and create populations that are better able to tolerate future conditions under climate change (Johnson and Munshi-South 2017).

Here, we identify potential positive impacts of cities on regional ecosystems. We acknowledge the well established and overall negative consequences of cities on biodiversity (McDonald et al. 2020). We focus on highlighting specific ways that cities support plant and animal species while also examining the many negative impacts of urbanization. First, we discuss what makes urban landscapes unique in a landscape context and the species-specific implications of the unique conditions found in cities. We then propose five pathways by which cities can contribute positively to their regions, including providing release from pressures faced in the surrounding landscape, increasing regional habitat heterogeneity, providing stopover habitat for migratory species, contributing to species genetic diversity and adaptation to climate change, and enabling and bolstering engagement and environmental stewardship. Our aim is to provide evidence for how, under what conditions, and for which types of species cities can have positive impacts in order to lay the groundwork for identifying urban conservation actions with greatest potential to be effective.

## What makes cities unique in their landscapes?

**Figure 1.**
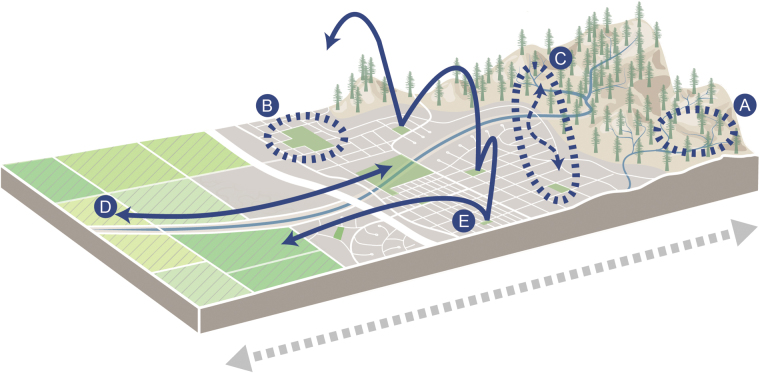
Abiotic and biotic differences between cities and their surrounding landscapes create strong gradients (grey arrow) in environmental and physical conditions. These gradients result from changes caused by urbanization and underlying conditions related to where cities are located. Arrows do not imply directionality in the difference between cities and their landscapes, which differ depending on locally specific conditions and on the type of gradient. Animals and plants respond to gradients in species-specific ways (blue arrows). Some species (a) are urban avoiders with populations that are primarily restricted to landscapes outside cities, (b) actively select urban areas, with populations primarily contained within city boundaries, (c) have large populations that span the urban boundary, (d) have large home ranges in which individuals move across the urban–rural gradient, and (e) are migratory and use the city or the surrounding landscape as stopover sites. Artwork by Katie McKnight.

Many cities are located along coasts, at major estuaries, near inland waterways, and in alluvial valleys—locations that historically allowed people to take advantage of temperate climates, rich agricultural soil, and navigational opportunities (Kühn et al. 2004). As a result, more people live at lower elevations and within 100 kilometers of a shoreline than expected by chance (Luck 2007), and soil, topography, availability of freshwater, climate, and solar radiation all play a role in where contemporary cities are located (Kühn et al. 2004). The same factors that draw people to these areas also tend to support other taxa, and many cities have been built in biodiversity hotspots and in locations with high net primary productivity (Luck 2007). The result can lead to strong environmental gradients between cities and their surrounding landscapes in geology, topography, elevation, and hydrology that are unrelated to human modification or urbanization (figure 1).

Urbanization also alters abiotic and biotic conditions, creating gradients between cities and their surrounding landscapes in hydrology, air temperature, atmospheric chemistry, and climate (Grimm et al. 2008a, Pickett et al. 2011, Kaushal et al. 2014). For example, the urban heat island effect increases mean growing season lengths and shifts phenology in many plant species, leading to earlier and longer flowering seasons than in rural areas (Harrison and Winfree 2015, Leong et al. 2016). In addition, resources and nutrient availability are often altered in cities because of the presence of human food (including supplemental feeding and food waste) and differences in net primary productivity.

Differences between cities and their surrounding landscapes also depend on what type of land use surrounds a city. Although relatively intact habitat is found around some cities, many are surrounded by highly modified landscapes, either through intensive or extensive agriculture or plantation forests. How cities compare with their surroundings is highly context dependent, given that both cities and their surroundings vary globally in the degree of human disturbance and extent of modification. For example, in a recent analysis across three continents, cities with more vegetation showed less of a difference in the functional diversity of avian assemblages compared with their surroundings (Oliveira Hagen et al. 2017). Resource availability and habitat quality can also change over time as cities age and as human behavior and preferences shift, leading to shifts in species responses. In European cities, black-billed magpie (*Pica pica*) populations have grown dramatically in cities over the past five decades, likely in response to decreased persecution by humans and winter bird feeding (Jokimäki et al. 2017). These changes highlight that as external factors shift, species with traits tolerant of urbanization may respond by venturing into cities to use their resources more frequently.

## How do species interact with cities?

**Figure 2.**
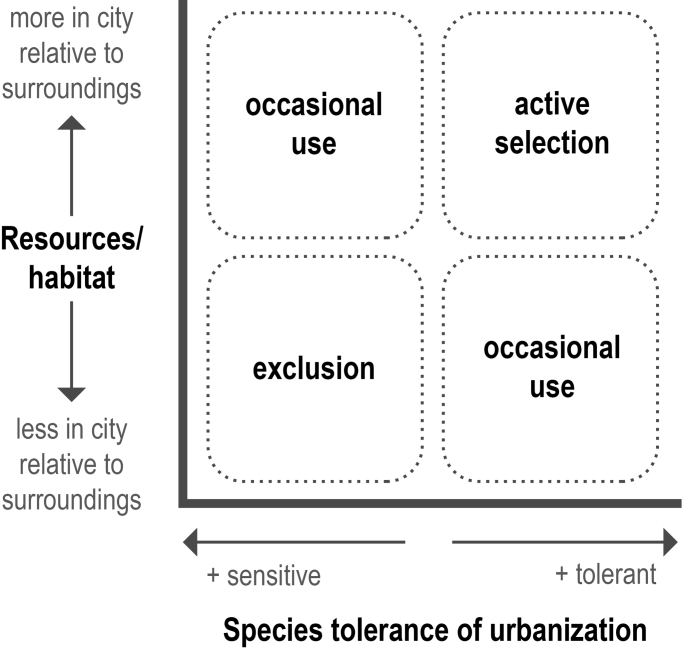
How frequently species are found in cities depends on a combination of species traits that either confer urban tolerance or sensitivity, and a response to the available resources and habitat in cities compared with the surrounding landscape. The landscapes around cities are highly variable in the degree of disturbance, habitat quality, and habitat heterogeneity, leading to variation in how cities compare with their surroundings in terms of available resources and habitat. Species that are tolerant of urbanization and for whom resources are more available in cities than elsewhere are likely to actively select urban habitats, whereas those that are sensitive to urbanization and find higher quality habitat in nonurban landscapes are likely to be nearly or entirely excluded from cities.

How species respond to urban landscapes depends on a combination of species traits, the relative availability of resources and habitat, and the presence of threats in cities compared with their surrounding landscapes (figure 2). Use of cities also depends on how species move across the landscape and the size of typical home ranges (see figure 1). Individuals with large home ranges may move back and forth across the urban–rural gradient. For example, tracking coyotes (*Canis latrans*) and red foxes (*Vulpes vulpes*) in Madison, Wisconsin, in the United States, revealed that both species’ home ranges spanned urbanized and natural areas (Mueller et al. 2018). In other species, such as great tits (*Parus major*) in Vezprem, Hungary, dispersal between urban and rural sites may be extremely limited, and individuals may be mostly restricted to either urban or rural areas (Seress et al. 2020). Some species also have migratory behavior that includes cities as stopover sites. In Sacramento, California, in the United States, for instance, migratory songbirds use valley oaks (*Quercus lobata*) as stopover sites in a matrix of residential backyards and woodlands (Greco and Airola 2018).

Cities act as strong filters on biotic communities (Aronson et al. 2016), and across taxonomic groups, species traits have been found to be related to urban tolerance. There is also evidence for phylogenetic signals across several taxonomic groups, indicating that more closely related species tend to respond similarly to urbanization (Callaghan et al. 2019, Winchell et al. 2020). Traits associated with urbanization include large litter size, higher brain mass, and diet diversity in mammals (Santini et al. 2019), adaptations for dry conditions and locomotion in urban environments in *Anolis* lizards (Winchell et al. 2020); generalist diet, large niche or habitat breadth, large clutch size, aboveground nesting, and large brain size in birds (Evans et al. 2011, Sol et al. 2014, Coetzee et al. 2018, 2019, Callaghan et al. 2019, 2020, Sayol et al. 2020); adaptation to open and edge space foraging and flexible roosting strategies in bats (Jung and Threlfall 2018); and smaller body size, more trait variation, and bimodality in tongue length in bumblebees (Eggenberger et al. 2019). In plants, nutrient-demanding traits and preference for drier to mesic soil conditions, human-assisted dispersal, and trees with showy reproductive parts have all been found to be more common in cities (Jenerette et al. 2016, Kalusová et al. 2016), although a recent review found little evidence of consistency across studies in plant traits associated with urbanization (Williams et al. 2015).

Variable species traits and local conditions lead to large differences in how species occupy and move through cities. Some species avoid cities completely, whereas others actively select urban landscapes (figure 2). The majority of species lie somewhere between these two extremes, although most are more common outside of cities. For example, in a global analysis of 27 cities including 1036 bird occurrence records, only 35% were more common in urban environments than in nonurban (Sayol et al. 2020). In another similar analysis including 529 bird species from three continents, 27% of species were restricted to nonurban areas, whereas only 12% were restricted to cities (Oliveira Hagen et al. 2017). In a global study of birds, species loss due to urbanization varied from 19.5% to 76.5%, with more extreme loss in the most urbanized locations (Sol et al. 2014). Of around 500 species across 11 taxonomic groups of mammals globally, between 1.9% and 20% of species were classified as either urban visitors or urban dwellers (Santini et al. 2019).

**Figure 3.**
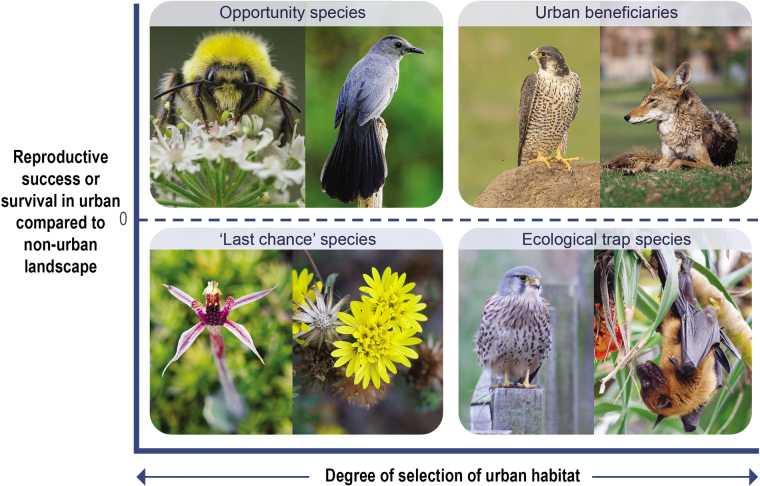
Among plant and animal species that use cities, there is variation in the degree to which they actively select urban habitat, and in their reproductive success or survival in cities compared with surrounding landscapes. “Last chance” species, such as the San Francisco lessingia (Lessingia germanorum) and Canberra spider orchid (Caladenia actensis), may not actively select or have higher reproductive success in urban landscapes, although cities may also represent their only remaining chance for survival (Soanes et al. 2019). Species that actively select cities despite lower reproductive success, such as the Eurasian kestrel (Falco tinnunculus) and the Indian flying fox (Pteropus giganteus), may be falling into an “ecological trap.” Species that actively select and have higher reproductive output in cities can be considered urban beneficiaries, including coyotes (Canis latrans) and peregrine falcons (Falco peregrinus). Opportunity species, such as the buff-tailed bumblebee (Bombus terrestris) and gray catbird (Dumetella carolinensis), may have high reproductive success without necessarily actively selecting urban habitats. Photographs: (clockwise from top left) David Marquina Reyes, N. Lewis/NPS, Hari Patibanda, Dru Bloomfield, Tobias Hayashi, Will Elder/NPS, Alastair Rae, and Jan Arendtsz.

The mere presence of a species in a city does not indicate that the species prefers urbanization, or that the species experiences higher reproductive success or survival in urban environments (figure 3). For example, in Veszprem, Hungary, urban great tit nestlings were found to be smaller and have lower survival rates than rural conspecifics, and a food supplementation experiment demonstrated that a lack of sufficient insect prey during the breeding season was likely responsible (Seress et al. 2020). In some species, there is evidence that urban habitats can create ecological traps, where individuals preferentially select what appears to be high quality habitat, only to face higher mortality or poorer resource availability in those locations (Battin 2004, Spear 2018). In Vienna, Austria, the Eurasian kestrel (*Falco tinnunculus*) has higher breeding densities in the city center, but lower reproductive success compared with suburban breeding individuals (Sumasgutner et al. 2014). Authors suggest that the species may face a trade-off between the availability of nesting sites and poorer prey availability in the urban center, suggesting the species may be falling into an ecological trap. Similarly, Indian flying foxes (*Pteropus giganteus*) are attracted to exotic fruiting trees in cultivated in gardens where they are also vulnerable to high rates of electrocution from nearby power lines in Sri Lanka (Tella et al. 2020). With the exception of the above examples, whether cities constitute ecological traps is unknown for most species. Although a species may be abundant in urban sites, abundance alone may mask low survival or poor breeding performance, leading to false conclusions about the potential benefits of cities to regional populations (Demeyrier et al. 2016, Kettel et al. 2018). Therefore, the information needed to understand the complex implications of urbanization on population dynamics remains unavailable for many species.

Species vary in the degree to which they actively select urban landscapes and in their reproductive success and survival in urban compared with nonurban landscapes (figure 3). Some species actively select urban landscapes. For example, in a recent analysis of 529 bird species globally, 66 species were found exclusively in urban areas. Beyond classic urban exploiters, this group included a range of species native in their region such as the mallard (*Anas platyrhynchos*), peregrine falcon, burrowing owl (*Athene cunicularia*), hooded crow (*Corvus cornix*), bronzed cowbird (*Molothrus aeneus*), and black-and-rufous warbling finch (*Poospiza nigrorufa*; Oliveira Hagen et al. 2017). Of species that actively select urban landscapes, some may be urban beneficiaries with high reproductive success or survival in cities compared with rural landscapes, such as peregrine falcons (*Falco peregrinus*; Kettel et al. 2018), raccoons (*Procyon lotor*; Prange et al. 2003), several bird species (Chamberlain et al. 2009), and coyotes (Prange et al. 2003).

The majority of species do not actively select urban habitats. For some species, cities have been built on their primary habitat, and they may persist in remnant urban habitat patches, where their reproductive output may be low compared with before urbanization. In Australia, for example, researchers recently identified 39 threatened “last chance” species of plants and animals (including orchids, trees, shrubs, a tortoise, and a snail) that are restricted entirely to small urban habitat patches (figure 3; Soanes and Lentini 2019). However, there are also some species whose reproductive success is higher in urban compared with nonurban landscapes, although they may not actively select cities. These species may represent an opportunity, and may be especially able to benefit from interventions to improve their habitat in urban areas (figure 3). For example, buff-tailed bumblebees (*Bombus terrestris*) were found to have higher reproductive success in urban sites compared with surrounding agricultural sites, although there was little evidence of active selection of urban sites (Samuelson et al. 2018). Similarly, higher survival in urban compared with surrounding forested sites was found in four species of birds including gray catbirds (*Dumetella carolinensis*), despite higher abundance in forested sites and little evidence of active selection of urban sites (Evans et al. 2015).

## How can cities benefit regional ecosystems?

Drawing on a range of examples from around the world, we identify five primary categories of benefits cities can provide to species (figure 3). Each category is described in more detail below.

### Cities can provide release from pressures faced in the surrounding landscape

Cities can buffer regional plant and animal populations during periods of stress and scarcity by providing altered or additional food and water resources not available in the surrounding landscape. For instance, American black bears (*Ursus americanus*) in Colorado, in the United States, moved into urban areas during food-poor years and out of them during food-rich years (Baruch-Mordo et al. 2014). In India, Hanuman langurs (*Semnopithecus entellus*) suffered a massive die-off in an exurban wildlife sanctuary during an El Niño Southern Oscillation-related drought event. Adjacent urban populations in the city of Jodhpur were supported through the drought by irrigated vegetation and human feeding, and suffered no corresponding die-off (Waite et al. 2007). Birdfeeders can increase avian survival over winter when food is scarce in the surrounding landscape (Siriwardena et al. 2007, Fuller et al. 2008, Jones and Reynolds 2008, Schoech et al. 2008, Plummer et al. 2019). Similar findings have also been documented for overwintering striped skunks (*Mephitis mephitis*) in Canada (Rosatte et al. 2011), and red kites (*Milvus milvus*) undergoing species recovery in the UK (Orros and Fellowes 2015).

Management of urban vegetation can alter both productivity and phenology, which can also make resources available at unique times. For example, ornamental plantings with long season blooms, urban warming, irrigation in arid environments, and tree removal in temperate environments can all shift flowering phenology. These changes can act as a filter, selecting against pollinators with phenology adapted to closely match native plants. However, some species may extend their flight seasons to forage in different habitats across the urban–rural gradient (Harrison and Winfree 2015). Evidence of this phenomenon comes from California, United States, where two frequently collected bee species were found most often in the early spring in natural sites, and in urban areas during the summer, suggesting urban areas are supporting longer flight seasons, and that bees are tracking temporal variation in resources across the urban–rural gradient (Leong et al. 2016).

Altered conditions in cities can lead to changes in trophic interactions that can free up resources and increase prey density. For example, higher prey density and reduced threat of persecution in cities has been linked to success in several urban raptors, including Cooper's hawks (*Accipiter cooperii*), peregrine falcons, crested goshawks (*Accipiter trivirgatus*), and Mississippi kites (*Ictinia mississippiensis*; Parker 1996, Cava et al. 2012, Millsap et al. 2013, Kettel et al. 2018, 2019, Sumasgutner et al. 2020). However, abundance does not necessarily correspond to higher reproductive success, and raptors with the greatest reproductive performance in urban compared with rural landscapes consume birds instead of mammals, which are often scarce and nocturnal in urban environments (Kettel et al. 2019). Higher prey density has also been linked to native spider persistence in Sydney, Australia (Lowe et al. 2016).

Urban conditions can also release some species from interspecific interactions including competition, predation, herbivory, and parasitism. Coyotes, red foxes, and urban tolerant birds have all been found to escape from competition in urban landscapes in the United States (McKinney et al. 2011, Moll et al. 2018), and grasslands birds have lower rates of nest predation and brood parasitism in Illinois, in the United States (Buxton and Benson 2015). Additional evidence for release from predation has been found in Burrowing owls (*Athene cunicularia*) in Argentina (Rebolo-Ifrán et al. 2017), and in striped field mice (*Apodemus agrarius*) in Poland (Łopucki and Kiersztyn 2020). Escape from predation and competition has also been hypothesized, but not tested, in termites (*Coptotermes gestroi*) in Southeast Asia (Zhang and Evans 2017), and escape from competition with larger zooplankton has been hypothesized to explain success of smaller zooplankton in urban ponds in Belgium (Gianuca et al. 2018). Deciduous trees of 11 species have been found to escape from herbivory because of higher predation of insects by birds in 16 European cities (Kozlov et al. 2017). Finally, a recent meta-analysis showed that although some species exhibit poorer health in urban areas because of exposure to toxins and greater parasite loads, some taxonomic groups exhibit on average better body condition (mammals) and less parasitism (mammals and birds) in urban compared with rural areas (Murray et al. 2019).

### Cities can increase regional habitat heterogeneity

In some cases, their unique location can lead cities to have higher habitat heterogeneity than their surroundings (figure 1). For example, cities in Germany were found to have higher geological richness and to be more often located along navigable rivers than their surroundings. These environmental gradients were associated with greater environmental heterogeneity and higher native plant richness than surrounding landscapes (Kühn et al. 2004). Strong environmental gradients can also lead to the persistence (or potential for recovery) of rare habitat types that increase regional habitat heterogeneity and support rare species, harboring biodiversity that is either uncommon or absent in the surrounding landscape. In some cases, cities have been built in regional hotspots of habitat heterogeneity, and the unique species found there are likely to have suffered from habitat loss that occurred during urbanization. Where they remain, small patches of habitat within the urban landscape may be the only place where some local endemics persist, and although cities have overall negative impacts on these “last chance” species, they may also represent their only remaining chance for conservation (see figure 3; Soanes and Lentini 2019). Examples of this phenomenon can be found in the San Francisco Bay Area, United States, where unique geology, topography, and microclimates lead to high rates of endemism. In the city of San Francisco, United States, regionally rare coastal dune scrub habitat historically supported several locally endemic species, including the San Francisco lessingia (*Lessingia germanorum*), the Mission Blue (*Icaricia icarioides missionensis*), and the San Bruno elfin (*Callophrys mossii bayensis*) butterflies (Longcore et al. 2010). All of these species are rare or threatened, persisting only in regionally rare habitat remnants in the city and making unique contributions to the biodiversity of the region.

**Figure 4.**
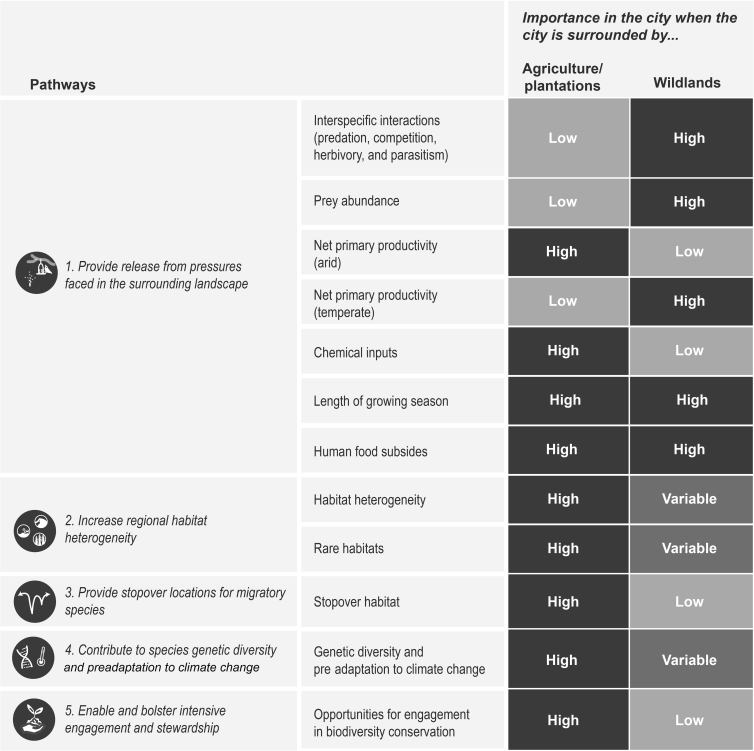
Cities provide habitat and increase population success for some species, with implications for regional biodiversity conservation. The positive contribution of cities can be grouped into five main pathways, each of which includes several factors that vary between urban and nonurban landscapes, depending on what land cover surrounds the city. For example, lower predator densities in cities compared with the surrounding landscape can release some species from predation in urban habitats, although this effect may be most pronounced in cities surrounded by wildland areas with large predator populations. Each cell shows the hypothesized importance of each factor in the city when the city is surrounded by either agricultural/plantation or wildland landscapes. Highly modified landscapes around cities can include intensive and extensive agriculture, or plantation forests.

Cities may also have higher habitat heterogeneity than their rural and exurban counterparts if the surrounding landscapes have been highly homogenized through agriculture, biological invasions, or other modifications or disturbance (figure 4). In Europe, agricultural intensification combined with conversion of some farmland back to forest has caused a decline in open woodlands with short-stature vegetation in landscapes surrounding cities. Consequently, the sparse trees and lawns found in moderate-density urbanized landscapes have become increasingly important as an alternative habitat for the common redstart (*Phoenicurus phoenicurus*), a species of conservation concern (Droz et al. 2019). In Australia, where 99% of the critically endangered Natural Temperate Grassland of the Victorian Volcanic Plain has been intensively cleared, a system of urban reserves in the city of Melbourne protects remnant patches of this rare habitat type, along with 234 native plant species (Kendal et al. 2017). A recent global review suggests pollinators may be doing better in cities, where they face less habitat homogenization and chemical exposure and can access more foraging and nesting resources, compared with agricultural landscapes (Hall et al. 2017). In southeast England, captive-reared colonies of wild-caught bumblebees (*Bombus terrestris*) had higher reproductive success in urban sites compared with agricultural sites (Samuelson et al. 2018). Similar evidence has also been found in Switzerland, where urban landscapes supported a higher abundance of bugs, beetles, and spiders, and higher species richness of bugs compared with intensively managed agricultural ecosystems (Turrini and Knop 2015).

Urbanization itself can also create heterogeneity in habitat conditions. Although habitats in cities tend to be highly fragmented, the variety in types of green spaces is diverse, including green roofs, vacant lots, street trees, managed public parks, forest remnants, and backyard gardens, all with varying management and species composition (Aronson et al. 2017). This diversity creates high fine-scale heterogeneity that can benefit some species. For example, in Chicago, Illinois, in the United States, networks of suburban yards along 1-kilometer transects with a mix of evergreen, deciduous, and berry-producing trees had higher species richness of birds than networks with more homogeneous tree cover (Belaire et al. 2014). Similarly, the diversity of garden features and gardening styles was associated with higher nesting density in bumblebees (*Bombus spp.*) compared with grasslands and woodland countryside habitats in the UK (Osborne et al. 2008). In San Francisco, California, in the United States, urban white-crowned sparrows (*Zonotrichia leucophrys*) had more diverse gut microbiomes than rural sparrows, likely because of higher land cover diversity and variation in tree cover in urban sites compared with rural sites dominated by a single shrub habitat type (Phillips et al. 2018). In a global analysis of 529 bird species, when correcting for species richness, functional diversity of birds was found to be higher in cities than in surrounding landscapes. This surprising result was attributed to higher habitat diversity in cities compared with the surrounding seminatural and agricultural areas that tended to support only a single habitat type (Oliveira Hagen et al. 2017).

### Cities can be used as stopover sites on migration routes

Cities can serve as stopover sites for migrating animals, and in some cases, urban stopover sites may contain denser food resources and fewer predators than sites outside cities. Cities may not necessarily be preferred over nonurban stopover sites, but may be used if the surrounding landscape is also highly altered, or if cities are located in unique locations along migratory routes. In both cases, alternative locations may be lacking, and species may continue to stop over in cities even though undisturbed habitat would be preferable. One example is New York City, New York, in the United States, which lies at the nexus of four major migratory flyways. Because much of the eastern seaboard of the United States is highly urbanized, city parks and other urban forest fragments and open space may represent the only available stopover habitat for many migrating birds. Large city parks, including Prospect Park in Brooklyn, New York (237 ha), support over 100 species of migrating songbirds (Seewagen et al. 2011) and over 200 species annually (La Sorte et al. 2020). Despite their much smaller area and higher densities of birds, urban parks were found to provide equivalent refueling capacity in 10 bird species compared with rural areas because of very high insect abundance (Seewagen et al. 2011). Evidence from large parks in several other cities, including Swainson's thrushes (*Catharus ustulatus*) in Highbanks Park (15.5 ha) Columbus, Ohio, in the United States (Matthews and Rodewald 2010), and four thrush species in a natural area (120 ha) in Detroit, Michigan, in the United States (Craves 2009), also suggest that large urban green spaces can act as important stopover locations for migrating songbirds.

### Cities can contribute to species' genetic diversity and preadaptation to climate change

Substantial evidence suggests that adaptation to urban environments, and genetic differences between urban and rural populations, are occurring in many species (Johnson and Munshi-South 2017). It is likely that these differences are affecting population genetics at the regional scale in both negative and positive ways. Despite this potentially important phenomenon, few studies have considered the implications of urban genetic change for regional populations. Although urban populations often exhibit greater genetic differentiation among subpopulations, lower genetic diversity, and reduced gene flow (Johnson and Munshi-South 2017), some species have higher gene flow in the city relative to rural populations (Miles et al. 2018).

A few urban-tolerant species have been found to have either higher genetic diversity within urban populations or little differentiation between urban and rural populations. For example, great tits had higher genetic variation in urban parks relative to surrounding forests, as well as evidence of gene flow from the city to the forest (Björklund et al. 2009). Black widow spiders (*Latrodectus hesperus*) in the western United States (Miles et al. 2018) and red-tailed bumblebees (*Bombus lapidarius*) in Germany (Theodorou et al. 2018) had higher genetic diversity, lower genetic differentiation, and higher genetic connectivity between urban compared with rural sites. Black-headed gulls (*Chroicocephalus ridibundus*) in northern Poland show evidence of extensive gene flow and little differentiation between urban and rural populations, likely because of the high dispersal ability, colonial life history, and migratory behavior of the species (Indykiewicz et al. 2018). These results suggest that mobile and urban-tolerant species may benefit from the contribution of cities to regional genetic diversity, without suffering the negative impacts caused by genetic drift.

A smaller number of studies have also documented adaptation and directional selection to urban environments (Johnson and Munshi-South 2017, Santangelo et al. 2018). In these cases, the contribution of urbanization-adapted individuals may increase genetic diversity at the landscape scale. Although it is possible that this could lead urban environment–adapted individuals to be maladapted to their surrounding landscapes (Spear et al. 2018), greater genetic diversity is also associated with an increased capacity to withstand environmental change, can be related to higher productivity and fitness, and can have cascading community effects that benefit other species (Hughes et al. 2008).

Adaptations to higher temperatures in urban areas have the potential to create populations that may be better able to tolerate future conditions caused by climate change and could act as source populations for colonization of rural areas in the future (we refer to this phenomenon here as *preadaptation* to climate change). A few recent studies document adaptations that confer tolerance to the hotter conditions found in cities. Urban populations of lesser pepperwort (*Lepidium virginicum*) in the northern United States bolt earlier and have a longer period between bolting and flower production—adaptations that are beneficial in hotter drier conditions and under water stress (Yakub and Tiffin 2017). Urban acorn ants (*Temnothorax curvispinosus*; Diamond et al. 2017) and water fleas (*Daphnia magna)* have higher heat tolerance than their rural counterparts (Brans et al. 2017). These results show evidence of adaptation that could help species cope with climate change, particularly if future climate conditions are similar to current conditions in cities.

### Cities can enable and bolster intensive engagement and stewardship

The close proximity of people to nature in cities creates opportunities for public engagement through education, citizen science, and stewardship programs (Soanes and Lentini 2019). Public engagement has helped support the recovery of peregrine falcons in cities and provided tangible opportunities for the public to assist in ongoing protection of an endangered species without leaving the city (Pagel et al. 2018). The proliferation of webcams enabling public viewing of nesting peregrines has promoted learning and empathic attitudes toward urban birds (Pagel et al. 2018) and curriculum programs centered around peregrine falcons introduce nature in the city to K–12 students around the United States.

Management for particular species or habitats provides hands-on stewardship opportunities to urban residents. Monarch butterflies (*Danaus plexippus*) are experiencing severe population declines in North America. Numerous organizations are promoting the planting of milkweed (*Asclepias* spp.), the Monarch caterpillar's host plant, in private gardens (Geest et al. 2019). These citizen scientist programs have attracted thousands of participants, and over 26,000 monarch waystations (managed gardens containing milkweed and nectar plants) were registered with MonarchWatch as of October 2019 (www.monarchwatch.org). Planting of native milkweed in butterfly gardens has been found to be an effective strategy. In Omaha, Nebraska, in the United States, for instance, similar recruitment, survival, and parasitism was found for Monarch butterflies in tall grass prairie conservation areas and suburban gardens, suggesting that private yards with milkweed maintained for Monarchs can contribute to their conservation (Geest et al. 2019). Widespread planting and management of milkweed may be more feasible in private gardens than in large public open spaces and parks, where active restoration programs often face challenges of obtaining and retaining funding, staffing, and project sustainability (Borgström et al. 2016).

In another example from the United Kingdom, urban ponds are managed by local residents (often a pond warden), and ponds are managed to support a wide range of successional stages. These actions can promote biodiversity, and urban ponds support similar alpha diversity of aquatic macroinvertebrates, as well as higher prevalence of some taxonomic groups than nonurban ponds (Hill et al. 2017). These management activities are made more feasible because of the ease of access that is possible in cities and can feed back to create more engaged community members with greater incentives to engage in stewardship actions (Mumaw 2017).

## Conclusions

Cities can benefit some species by releasing them from threats in the larger landscape, increasing regional habitat heterogeneity, acting as migratory stopovers, enhancing regional genetic diversity and providing selective forces for species to adapt to future conditions under climate change (e.g., a phenomenon we are calling *preadapting* species to climate change), and enabling and bolstering public engagement and stewardship (figure 4). Although most species are negatively affected by urbanization, cities also produce a unique set of resources that can buffer some species during periods of scarcity and provide release from threats faced outside cities. The benefits of cities to species vary widely in their implications for reproductive success, survival, and long term conservation potential. For example, urbanization has an overall negative impact on “last chance” species, although cities may represent their only remaining chance for conservation (Soanes and Lentini 2019). Other species actively select urban landscapes, and some have higher population growth rates in cities compared with their surroundings. Many species make use of urban landscapes, but may not actively seek them out, including migratory species that use cities as stopover habitat and species that move in and out of urban landscapes during periods of stress.

Responses to urbanization are highly species-specific and depend on a combination of species traits and the characteristics of both the city and its surrounding landscape. In many cases, species responses to cities have changed over time as the habitat and resource context shifts. This implies that actions that we take focused on urban biodiversity conservation can broaden the suite of species that are able to take advantage of the resources cities have to offer while reducing their negative impacts. Although a small subset of species clearly benefit from cities, for many, the true population and demographic outcomes of urban living remain unclear. A recent meta-analysis of urban raptors found that several species that are more abundant in urban landscapes compared with rural have lower reproductive success in cities, highlighting that abundance alone is a poor proxy for gauging the population-level implications of cities (Kettel et al. 2019). Although many studies have compared reproductive rates and other demographic parameters in and outside of cities, there remain many species for which the true effects of urbanization remain poorly understood.

An expanded research agenda could fill this gap, deepening our understanding of the ways cities can support regional biodiversity conservation and providing information to guide the planning and design of urban green spaces. For example, urban greening actions can be informed by design guidance derived from biodiversity-focused research in cities (Spotswood et al. 2019). Broadening the scope of research to understand the role cities play in supporting regional populations of species that use urban habitat, could provide greater context, motivation, and support for these activities while also informing landscape-scale conservation actions. We hypothesize that cities may be increasing regional genetic diversity to the benefit of some species, and although this outcome can be logically inferred given the link between genetic diversity and population and community resilience (Hughes et al. 2008), we found no studies that directly addressed the question. This unexplored avenue for future research could shed light on the potential for urban adaptation to positively affect regional population genetics. Another potentially powerful avenue to pursue would be to link known adaptations to future climate projections outside cities.

Expanding our understanding of these positive impacts can help support a growing recognition that urban ecosystems are a necessary component of landscape-scale biodiversity conservation (Soanes and Lentini 2019) and should be part of a broader effort to reconcile anthropogenic habitats with biodiversity (Rosenzweig and Michael 2003). Including urban landscapes in the suite of locations where conservation is possible could expand conservation opportunities, opening up a range of urban greening-focused actions that could make positive contributions to regional biodiversity (Soanes et al. 2019). The majority of mechanisms identified in the present article are underrecognized, and the benefits to species happenstance rather than resulting from planned, coordinated conservation efforts. Increased recognition of these mechanisms could provide greater scientific guidance and a broader platform to motivate the integration of biodiversity conservation with other social goals, planning, and policy. Increasing conservation efforts in cities could also generate public interest in urban conservation, providing first hand experiences of habitat creation and positive interaction within natural systems; identifying these authentic stories is essential for cultivating an ethos of cultural and ecological sustainability (Kimmerer 2013). Engaging the public in meaningful urban biodiversity-focused activities also has the potential to increase public knowledge of and support for broader conservation initiatives, which will be critical to maintaining political and financial will for conservation in the future.

## Author Biographical

Erica N. Spotswood (ericas@sfei.org), Robin Grossinger, and J. Letitia Grenier are senior scientists at the San Francisco Estuary Institute in San Francisco, California in the United States. Erin E. Beller is the Urban Ecology Program manager for the Real Estate and Workplace Services Sustainability Team at Google, in Mountain View, California, in the United States. Nicole E. Heller is the curator of Anthropocene studies at the Carnegie Museum of Natural History, in Pittsburgh, Pennsylvania, in the United States. Myla F. J. Aronson is an assistant professor for the Department of Ecology, Evolution, and Natural Resources at Rutgers, The State University of New Jersey, in New Brunswick, New Jersey, in the United States.
